# A Highly Sensitive and Selective Isobutyraldehyde Sensor Based on Nanosized Sm_2_O_3_ Particles

**DOI:** 10.1155/2020/5205724

**Published:** 2020-04-01

**Authors:** Li Jiang, Yun Wu, Yan Wang, Qin Zhou, Yuguo Zheng, Yafei Chen, Qianchun Zhang

**Affiliations:** School of Biology and Chemistry, Key Laboratory of Chemical Synthesis and Environmental Pollution Control-Remediation Technology of Guizhou Province, Xingyi Normal University for Nationalities, Xingyi 562400, China

## Abstract

A highly sensitive and selective sensor for isobutyraldehyde (IBD) is demonstrated based on intensive cataluminescence (CTL) emission from the surface of nanosized Sm_2_O_3_ particles. The characteristics and optimum conditions for the CTL sensor, including the working temperature, wavelength, and flow rate, were investigated in detail. Under the optimized experimental conditions, the CTL intensity varied linearly with the concentration of IBD, in the two-order-of-magnitude range of 0.015–3.9 *μ*g/mL, with a correlation coefficient (*r*) of 0.99991 and a limit of detection (LOD), at a signal-to-noise ratio (S/N = 3) of 4.6 ng/mL. The sensor was quite specific: butyraldehyde, methanol, ethanol, acetone, formaldehyde, acetaldehyde, benzene, ethylbenzene, and cumene could not produce significant CTL intensities; specifically, butyraldehyde, ethanol, acetone, and acetaldehyde produced low CTL intensities, with values that were 3.8%, 2.8%, 0.60%, and 0.57% that of IBD. As a test of sensor stability, we found that the relative standard deviation (RSD) of 30 measurements of the CTL at an IBD concentration of 1.6 *μ*g/mL within a period of 72 h was 2.2%, indicating good stability and long service life of the sensor. The sensor was tested against spiked samples containing IBD, and recoveries between 89.7% and 97.4% were obtained with an RSD of 6.1%–8.6%. The performance of the sensor indicated its utility for practical sample analysis.

## 1. Introduction

Isobutyraldehyde (IBD) is an important organic chemical raw material. Starting from IBD, many high-value-added chemical products can be synthesized, such as isobutanol, isobutyric acid, acetal, oxime, and imine [1]. These products have immense market potential in many areas. For example, isobutanol can be substituted for gasoline or used as a chemical feedstock [2]. IBD is a volatile organic compound (VOC) that also has irritating and allergy-sensitizing characteristics. IBD can irritate the eyes, nose, and respiratory tract of the human body at low concentrations and can produce anesthetic effects at high concentrations. IBD gas is somewhat dangerous, as it is flammable and reacts strongly with oxidants. IBD vapor is heavier than air, leading it to become concentrated in depressions; further, it has high diffusivity, which raises the risk of its spreading rapidly and reigniting in case of fire. Therefore, the detection and recognition of IBD have emerged imperative from the standpoint of environmental protection and human safety. Although traditional methods for measuring IBD concentrations, such as gas chromatography, mass spectrometry, and optical spectrometers, have the advantage of high sensitivity, such instruments are often expensive, cumbersome, and time-consuming [3, 4]. Since Breysse et al. [5] first proposed cataluminescence (CTL), CTL-based gas sensors have been widely studied [6–8].

CTL is chemiluminescence due to a catalytic reaction that occurs on the surface of a solid catalyst, where the reaction is accompanied by chemiluminescent emission. CTL has the advantages of high sensitivity, rapid response, simple instrumentation, and low background signal levels [9]. To date, many different materials have been used to developed CTL-based sensors. Examples include a sensor for detecting formaldehyde and carbon monoxide based on Pt-activated Ce_4_La_6_O_17_ nanocomposites [10], an acetaldehyde gas sensor based on PdO-ZnO p-n heterojunction nanostructures [11], an acetone gas sensor based on mesoporous Mg-doped SnO_2_ structures [12], a diethyl ether sensor based on nanoparticles of TiO_2_ [13], an *n*-propanol gas sensor based on a composite of SrCO_3_/graphene [14], an H_2_S gas sensor based on enclosed hollow tubular ZnO [15], an acetophenone gas sensor based on nano-Pr_6_O_11_ [16], a benzene and toluene sensor based on TiO_2_/SnO_2_ [17], and a formaldehyde and ammonia gas sensor based on nano-Ti_3_SnLa_2_O_11_ [18]. The sensing material clearly plays a key role in a sensor system, as it directly affects the sensitivity, selectivity, and stability of the sensor.

Samarium (III) oxide (Sm_2_O_3_), an important rare-earth oxide, has been studied owing to its strong catalytic potential. Sm_2_O_3_ is a p-type semiconductor [19], with a behavior that is different from *n*-type semiconductors. This material has lower electrical conductivity at high temperatures and readily exchanges its lattice oxygen with the oxygen in the air. These advantages are important not only in maintaining the long-term stability of the sensor but also in extending its lifetime [20]. However, using nano-Sm_2_O_3_ as a CTL gas sensor is not reported, only a few studies have been reported to date on Sm_2_O_3_ for other sensors, such as Renganathan et al. [21] reported the development of a Sm_2_O_3_ fiber optic sensor for ammonia, methanol, and ethanol; Zhou [22] reported on the use of SnO_2_ decorated with Sm_2_O_3_ as a chemical sensor for acetylene; and Jamnani et al. [20] reported on a Sm_2_O_3_ conductometric sensor to be used for ethanol and acetone. We have not found any corresponding report on IBD sensors.

In this work, we have developed a novel IBD sensor based on CTL using nano-Sm_2_O_3_. Intense CTL emissions were observed when IBD was passed by the surface of particles of nano-Sm_2_O_3_. Results of further testing showed that this CTL sensor was not only highly sensitive but also highly selective and that it has the ability to detect low concentrations of IBD with the advantages of high response speed and good stability. The gas sensor developed in this study was tested by applying it to the determination of IBD in spiked samples.

## 2. Experiments

### 2.1. Instruments and Reagents

We used the BPCL-2 ultra-weak luminescence analyzer manufactured by the Institute of Biophysics, Chinese Academy of Science (Beijing, China), to detect and process the CTL intensity; the YZ1515x micro air pump we employed was manufactured by Bao Ding Chuang Rui Precision Pump Co., Ltd. (China), and it provided power and oxygen; the TDGC 2 voltage regulator purchased from CNCQIANG Electric Co., Ltd. (Zhejiang, China) provided the heater for the ceramic rod, and a GC/MS (Shimadzu QP-2010) was used to verify the results of using the CTL method.

All reagents used in our experiments were of analytical grade. Samarium (III) nitrate hexahydrate (Sm(NO_3_)_3_·6H_2_O), 3-nitrobenzoic acid (C_7_H_5_O_4_N), IBD, butyraldehyde, methanol, ethanol, acetone, formaldehyde, and acetaldehyde were purchased from Aladdin Chemical Co., Ltd. (Shanghai, China); benzene, ethylbenzene, and cumene were obtained from J&K Scientific, Inc. (Beijing, China).

### 2.2. Experimental Equipment

**Figure 1:**
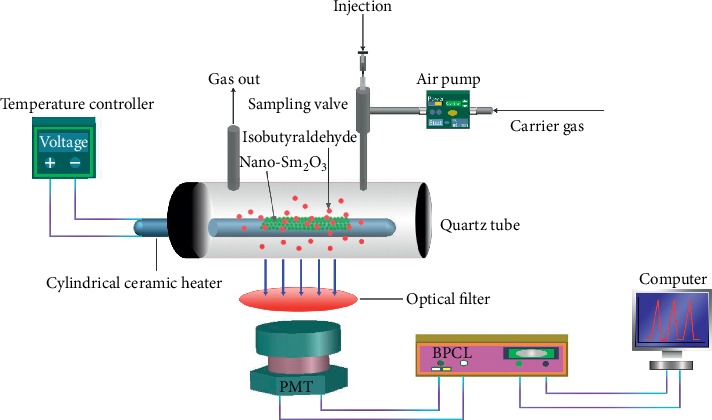
Schematic diagram of the CTL sensing system device.

The CTL experimental setup is shown in Figure 1. These are its principal parts: (1) power device: a micro air pump was used to provide power and oxygen; (2) sample injection device: a stainless steel three-way valve was used as the injection valve; (3) CTL reaction chamber: this consisted of a ceramic heating rod with nano-Sm_2_O_3_ sintered on the surface and a quartz tube with an air inlet and outlet. The ceramic heating rod was placed in the quartz tube; (4) temperature control device: we achieved control of the surface temperature of the ceramic heating rod by adjusting its working voltage; (5) spectroscopic device: this used interference filters with wavelengths of 412 to 520 nm (412, 425, 440, 475, 490, 505, and 520 nm). The appropriate analysis wavelength was selected to eliminate the background interference; (6) photoelectric detection and data processing system: this consisted of a BPCL-2 ultra-weak luminescence analyzer and computer for detecting and processing the CTL signal.

### 2.3. Detection Method

The temperature of the surface of the ceramic heating rod was adjusted and controlled to the temperature required for the reaction; control was achieved through the voltage regulator, following which we selected the appropriate detection wavelength and carrier gas flow rate. We used a gas syringe to draw a 1.00 mL sample of a known concentration after the gas had been injected into the system pipeline from the injection valve. The sample was carried by the air carrier gas into the CTL reaction chamber. It contacted the nano-Sm_2_O_3_, which was oxidized by oxygen on its surface and generated the CTL signal. After being processed by the data processing system, the CTL intensity signal was directly read and recorded by the computer.

### 2.4. Synthesis of Particles of Nano-Sm_2_O_3_

Nano-Sm_2_O_3_ was synthesized as follows: 2.4 g of Sm(NO_3_)_3_·6H_2_O and 2.4 g of C_7_H_5_O_4_N were each added to 30 mL of distilled water, and the two solutions were each ultrasonicated for 1 h. We mixed the two solutions together and added ethanol solution dropwise with magnetic stirring to maintain the pH at 5. Then, the solution was transferred into a Teflon-lined autoclave of 100 mL capacity, sealed, and maintained at 150°C for 3 h and then cooled to room temperature. The precipitate was separated by centrifugation, washed with distilled water and ethanol several times, and calcined at 600°C for 2 h.

## 3. Results and Discussion

### 3.1. Characterization of Nano-Sm_2_O_3_

**Figure 2:**
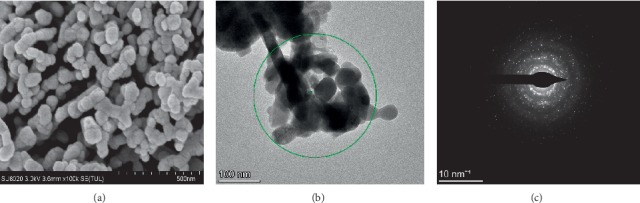
(a) SEM images of nano-Sm_2_O_3_; (b) TEM images of nano-Sm_2_O_3_; (c) SAED of nano-Sm_2_O_3_.

The morphology of the synthesized Sm_2_O_3_ particles was characterized using a field-emission scanning electron microscope (SU8020) and transmission electron microscope (JEM-1200EX).  Figure 2(a) shows the morphology of this sample at a magnification of 100,000x; the synthesized sample has a loose and porous structure that facilitated the contact of the test gas with the sensor surface and enhanced the CTL performance.  Figure 2(b) shows that the typical particle size of this sample was approximately 40 nm.  Figure 2(c), which shows the corresponding selected area electron diffraction pattern, further confirmed the crystalline nature of the sample [23].

**Figure 3:**
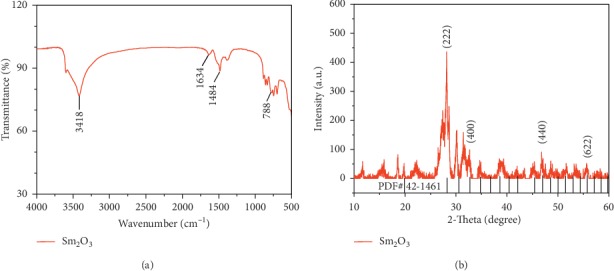
(a) FT-IR spectrum of nano-Sm_2_O_3_; (b) XRD pattern of nano-Sm_2_O_3_.

The Fourier-transform infrared (FT-IR) spectrum of nano-Sm_2_O_3_ in the range of 4000–500 cm^−1^ is shown in Figure 3(a). A sharp absorption band appeared at 3418 cm^−1^ due to the tensile vibration of the O–H group; simultaneously, it was confirmed that there is crystalline water in the crystal [24, 25]. The absorption at 1634 cm^−1^ is the result of the combined action of bending vibrations of water molecules and asymmetric tensile vibrations of C = O groups [26, 27]. The sharp absorption band at 1484 cm^−1^ was due to the tensile vibration of C=C [26]. The existence of Sm-O was confirmed by the peak observed at 788 cm^−1^, as the FT-IR spectrum analysis of nano-Sm_2_O_3_ confirmed the existence of related functional groups such as Sm-O [27].

The microstructure of the synthesized samples was studied by X-ray diffraction. The results are shown in Figure 3(b). The main diffraction peaks are present at 2*θ* = 28.254°, 32.741°, 46.978°, and 55.739°, which can be allocated to 222, 400, 440, and 622, respectively; these results are identical with the standard XRD patterns of Sm_2_O_3_ (PDF# 42-1461) [28].

### 3.2. Cataluminescence Response Performance

#### 3.2.1. Selectivity and Specificity

**Figure 4:**
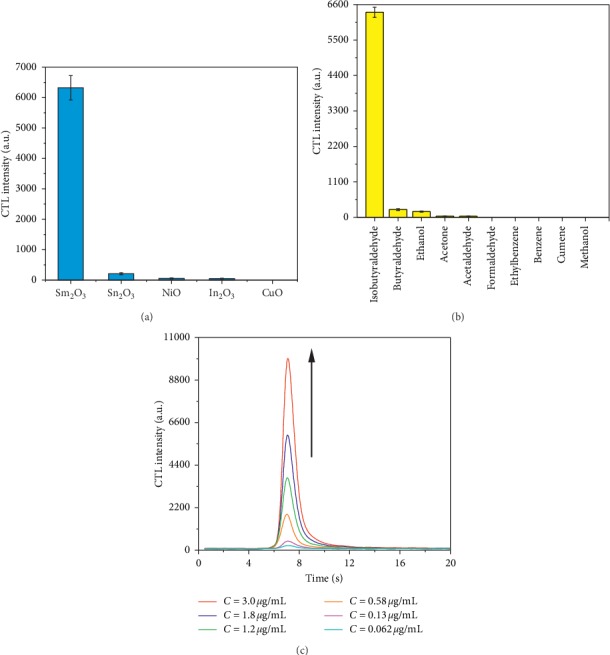
(a) CTL response to IBD by different materials; (b) CTL response to different VOCs by nano-Sm_2_O_3_; (c) CTL response curves versus time for different concentrations of IBD on nano-Sm_2_O_3_.

The sensitivity of a material, which is the response to the compound of interest, plays an important role for the sensor. To select an appropriate sensing material to design the sensor for IBD, the CTL emissions of IBD from nano-Sm_2_O_3_, nano-Sn_2_O_3_, nano-NiO, nano-In_2_O_3_, and nano-CuO were investigated. As shown in Figure 4(a), IBD produced the highest CTL response from the surface of nano-Sm_2_O_3_; however, the IBD intensity on the surface of nano-Sn_2_O_3_, nano-NiO, and nano-In_2_O_3_ was 3.30%, 0.89%, and 0.81% lower than that of nano-Sm_2_O_3_, respectively. There was no CTL response on the surface of nano-CuO. Therefore, nano-Sm_2_O_3_ was chosen for the subsequent experiments. In addition to sensitivity, specificity, which is the response to materials not of interest, also plays an important role in designing a gas sensor. CTL responses to emissions of IBD, butyraldehyde, methanol, ethanol, acetone, formaldehyde, acetaldehyde, benzene, ethylbenzene, and cumene on nano-Sm_2_O_3_ were studied. The results are shown in Figure 4(b): butyraldehyde, ethanol, acetone, and acetaldehyde each produced low CTL intensity, the values of which were 3.8%, 2.8%, 0.60%, and 0.57% lower than that of IBD, respectively. Other substances tested did not produce a CTL response. This indicated that the CTL sensor based on nano-Sm_2_O_3_ has good specificity to IBD.

#### 3.2.2. Response and Recovery Times

We measured the CTL response curves for different concentrations of IBD on the surface of nano-Sm_2_O_3_. Six different concentrations (0.062 *μ*g/mL, 0.13 *μ*g/mL, 0.58 *μ*g/mL, 1.2 *μ*g/mL, 1.8 *μ*g/mL, and 3.0 *μ*g/mL) of IBD had their CTL emission response signals studied in the subsequent experiments. The results are shown in Figure 4(c). From this figure, one notes that the CTL intensity of IBD on the surface of nano-Sm_2_O_3_ increased with the IBD concentration. The CTL response curves as functions of time for different concentrations were similar. For all six concentrations of IBD, the maximum signals were achieved after approximately 1 s after injection, which indicated a rapid response of the sensor to different concentrations of IBD. The recovery time of the sensor for IBD was approximately 6 s, demonstrating fast recovery by the sensor.

### 3.3. Optimization of Detection Conditions

**Figure 5:**
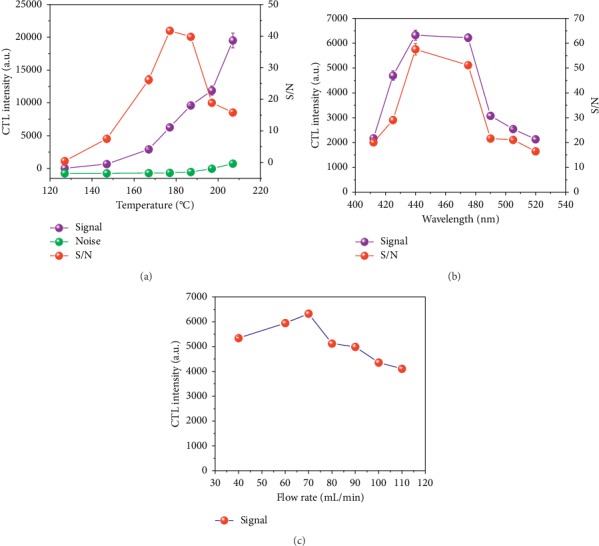
Effect of detection conditions on CTL intensity, noise, and S/N. (a) Effect of working temperature on CTL intensity, noise, and S/N; (b) effect of wavelength on CTL intensity and S/N; (c) effect of air flow rate on CTL intensity.

Figure 5 summarizes the effects of detection conditions on the CTL intensity, noise, and the signal-to-noise ratio (S/N). The wavelength, reaction temperature, and flow rate play important roles in the catalytic oxidation reaction of IBD on the surface of nano-Sm_2_O_3_. Each has a significant impact on the CTL intensity and S/N, in particular. We investigated the CTL intensity, noise, and S/N on IBD of concentration 1.6 *μ*g/mL, using nano-Sm_2_O_3_, with the response measured at a wavelength of 425 nm using a carrier gas flow rate of 120 mL/min.  Figure 5(a) shows the effect of working temperature on the CTL intensity, noise, and S/N; Figure 5(b) shows the effect of wavelength on the CTL intensity and S/N; Figure 5(c) shows the effect of air flow rate on CTL intensity. As shown in Figure 5(a), the CTL intensity, noise, and S/N curves of IBD were investigated in the range of 127–207°C; the noise mainly comes from heat radiation, it obviously increases at higher temperatures. In view of the S/N reaching a maximum at 177°C, 177°C was chosen as the optimal reaction temperature for the determination of IBD. As shown in Figure 5(b), we investigated the CTL intensity and S/N at several wavelengths and a carrier gas flow rate of 120 mL/min. The CTL intensity and S/N reached maxima at 440 nm. Therefore, 440 nm was chosen as the optimal analytical wavelength for the determination of IBD. The effect of carrier gas flow rate on the CTL intensity was investigated at a reaction temperature of 177°C and a detection wavelength of 440 nm. As shown in Figure 5(c), the CTL intensity of IBD is relatively low at lower flow rates, which may reflect the reaction-rate-control step under low carrier gas flow rates; however, at higher flow rates, the CTL intensity decreased. This may be because a higher carrier gas flow rate causes IBD to leave the catalyst surface with the carrier gas without effective oxidation on the catalyst surface. The flow rate of 70 mL/min was chosen as the optimal flow rate for the subsequent experiments because of the strong CTL emissions under this condition.

### 3.4. Calibration Curve and Reproducibility

**Figure 6:**
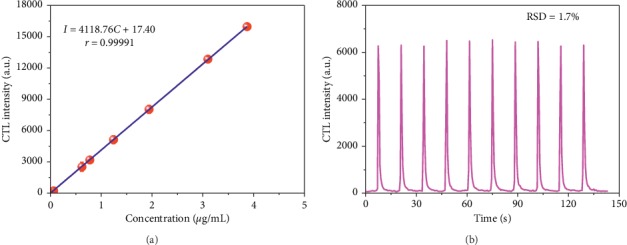
(a) Calibration curve between CTL intensity and concentration of IBD; (b) typical results obtained from ten replicate determinations of IBD within 150 s.

Under the optimal conditions, samples of the IBD gas at concentrations of 0.015 *μ*g/mL, 0.031 *μ*g/mL, 0.062 *μ*g/mL, 0.62 *μ*g/mL, 0.77 *μ*g/mL, 1.2 *μ*g/mL, 1.9 *μ*g/mL, 3.1 *μ*g/mL, and 3.9 *μ*g/mL were injected into the nano-Sm_2_O_3_ sensor, respectively. The calibration curve was drawn, as shown in Figure 6(a). There is a linear relationship between the CTL intensity and the concentration of IBD in the range of 0.015–3.9 *μ*g/mL. For Figure 6(a), the linear regression equation was characterized by *I* = 4118.76 C + 17.40 (*r* = 0.99991), where *I* is the CTL intensity and *C* is the IBD concentration. The LOD was found to be 4.6 ng/mL (S/N = 3). The relative standard deviation (RSD) for 10 detections of the CTL intensity at a concentration of 1.6 *μ*g/mL measured within 150 s was 1.7% (Figure 6(b)). In order to further study the lifetime of the sensor, we measured the RSD of 30 detections of the CTL intensity at a concentration of 1.6 *μ*g/mL within 72 h. The obtained RSD of 2.2% indicated good stability and long service life of the sensor.

### 3.5. Sample Analysis

To examine the practical application of the newly developed sensor for IBD, air samples that had been stored for long periods near the laboratory reagent cabinets were transferred to three sampling bags of 1 L volume. No CTL signals were detected for the samples by the sensor and GC/MS. The three stored air samples were then further spiked with IBD standards at three different levels, 0.0310, 0.310, and 3.10 *μ*g/mL. The results are shown in Table 1, which also identifies the other likely contaminants in the sampled air. The recoveries of IBD in the three samples were 89.7% to 97.4% with RSDs of 6.1% to 8.6%. These results indicated the potential of the CTL sensor for successful use in practical sample analysis.

## 4. Conclusion

In summary, we used synthesized particles of nano-Sm_2_O_3_ to develop a high-performance novel cataluminescence sensor for detecting isobutyraldehyde. Major detection parameters for the sensor included reaction temperature, detection wavelength, and carrier gas flow rate; these were systematically optimized for the determination of isobutyraldehyde. The sensor was successfully applied to the determination of isobutyraldehyde in spiked air samples. This gas sensor has the advantages of fast response, high sensitivity, satisfactory stability, good selectivity, and low cost. Therefore, our work has been developed and demonstrated a selective, sensitive, and convenient method for the rapid determination of isobutyraldehyde.

## Figures and Tables

**Table 1:** Analysis results of IBD samples measured by the proposed CTL sensor.

Sample no.	Composition	Spiked values (*μ*g/mL)	Measured values (*μ*g/mL)	Recovery (%)	RSD (%)
1	IBD	0.0310	0.0278	89.7	6.1
	Formaldehyde	0.0310			
	Ammonia	0.0310			
2	IBD	0.310	0.285	91.9	8.5
	Acetaldehyde	0.310			
	Ethanol	0.310			
3	IBD	3.10	3.02	97.4	8.6
	Acetone	3.10			
	Benzene	3.10			

## Data Availability

The data used to support the findings of this study are available from the corresponding author upon request.
